# Introductory Lecture to the Course of Medical Institutes in the University of Pennsylvania

**Published:** 1842-11-19

**Authors:** 


*Introductory Lecture to the Course of Medical Institutes in the University of Pennsylvania. Delivered November* 4, 1842. By SAMUEL JACKSON, M. D. Philadelphia: 1842. 8vo. p. 26.

This is a masterly exposition of the capabilities of our science. Professor JACKSON has always led, rather than followed in the path of medical educational improvement, and the present introductory lecture, affords abundant evidence of the same zealous and untiring spirit to point out the best means for the investigation of truth, as forming the fittest preparation to the pursuit of any branch of scientific inquiry. It is the production of a clear, intelligent and reflecting mind, and we trust will be deeply pondered on by those to whom it was addressed, and who have already evinced a just discrimination in requesting its publication.

After dwelling on the false doctrines which have alternately divided Medicine, Dr. J. observes, that though at the present day there is no general doctrine of Medicine, yet there are many special theories, limited and exclusive, which continue to be promulgated, and in illustration, aptly presents the theories of inflammation, as found in the surgical and pathological works of the day.

"The present doctrines of inflammation, are founded on the observations and experiments of Hastings, Kaltenbruner, Wilson Philip, Thompson, and especially Gendrin. They turn principally on supposed actions in the capillaries. The subjects of these experiments were cold-blooded animals, and the inferences were drawn from movements that were believed to take place in the capillary vessels, from motions seen in the blood corpuscles. But it is now established, and I have verified the fact by experience, that these animals are not susceptible of inflammation and suppuration. Inflammation cannot be produced in their tissues. The phenomena observed in them could not, therefore, resemble those of inflammation occurring in warm-blooded animals. It is clear, that the essential characters of inflammation, an affection that belongs to warm-blooded animals, could not be discovered, by the experiments that were made on cold-blooded animals for that purpose. In all these experiments, a most important circumstance was overlooked. An action of capillaries, contraction or dilatation, was inferred, for no such movements could ever be detected in the vessels themselves, from the movements that were observed in the blood corpuscles. But the agents employed in the experiments, are most of them capable of acting chemically on the blood corpuscles, which have a certain innate capacity of action. These active agents, when applied to the delicate, transparent tissues, are instantaneously mingled with the circulating fluid by endosmose, and thus brought into direct contact with the blood corpuscles. The motions observed in them, are the result of this direct action, between them and the chemical or other agents of the experiment. These movements are entirely independent of the capillary vessels, in which the blood-corpuscles are contained with the transparent blood. In some instances, as when ammonia is employed, the coloring matter of the blood-disk is separated, escapes by exosmose, and it dyes the tissue, without the slightest arrest, or any other change in the circulation.

"Other oversights, it is not necessary now to dwell on, were committed. These are sufficient to show, that the present received doctrines of inflammation, are founded on a basis, not only too narrow and exclusive, but actually a false one. If we do not, therefore, entirely reject them, we must, at least, look on them with suspicion, and hold them in distrust.

"Gendrin, whose observations are the most particular, and which are considered the most conclusive, and to be relied on, must have permitted his imagination to delude his perceptions, as he minutely describes to have seen, what is now demonstrated never could have occurred. He represents, as having witnessed passing under his eye, the conversion of blood corpuscles into pus-globules, in the web of a frog's foot, and the mesentery of a frog. Here are two palpable errors, or rather impossibilities. First, inflammation cannot be produced, nor pus formed in frogs. Second, the pus-globule is never formed from blood corpuscles, but from exudation cells, that are never thrown out in that class of animals."

The claims of organic chemistry are ably investigated, and impartially and fairly decided on.

With not a few, chemistry is outlawed from the domain of vitality. Because the chemical phenomena of living beings, are not the chemical actions of inorganic bodies; because the chemical processes of the living organism, cannot be imitated in the processes of our laboratories, they are denied to be of a chemical nature. If combinations of chemical elements and new arrangments of chemical equivalents; if the changes of component atoms, productive of new forms and new properties, be chemical, and I know not what other is chemistry, then, the great organic functions, respiration, digestion, secretion, nutrition, must be essentially chemical actions, regulated and determined by the laws of vitality.

The two proximate facts of vital activity, persisting from the first to the last movements of life; that follow them through all their grades and modifications, indicating their force and condition, are the combination of the oxygen of the atmosphere with the carbon of the organic matter, and the consequent development of temperature. Surely, if phenomena can be regarded as chemical, these must belong to that class of facts.

A stronger illustration is presented in the development of the chick in the egg. Albumen, some animal oil, and a microscopic germinal spot or vesicle, are the contents of the egg. During incubation there is no addition but oxygen from the atmosphere; nothing is given off but carbon. Yet there is formed, by the vital chemistry of this organic laboratory, blood, membrane, muscles, nerves, glands, secretions, bone, feathers, horny substance. The chemical elements, and, as respects the most important tissues, the chemical equivalents of the original albumen, the organizable substance, are precisely the same, as the chemical elements and chemical equivalents of these varied and dissimilar organized structures. The only modification has been a new arrangement of the chemical equivalents of the albumen, or the loss of some atoms of its carbon, and addition to it of some atoms of oxygen. Here is presented the strongest evidence of chemical actions.

Chemical actions are, however, but a part, a very subordinate part, of the important phenomena of vitality. The creative force of life has used chemical laws for the composition of the materials it employs, in the production of forms, the construction of its instruments, or organs, and the creation of a living being, after especial types. Chemical actions are means to produce an end: they are agents, assisting in the organization of living tissues, but can never of themselves produce organization, not even of the simplest structure.

Not less striking is the evidence furnished by the successive transformation of the aliment, vegetable and animal, into chyme, chyle and blood. The chemical equivalents, of those different substances are the same as to number and weight. Yet how different are these substances, in all their properties. The blood, the organic fluid, is transformed into solids, muscle or flesh, and nerve substance. But no change in the chemical elements or equivalents has been necessary. The chemical equivalents of muscle, are precisely the same, as to number and weight, as those of the aliment and animal food and nitrogenized vegetables. Little as these various substances resemble one another, there has not been the addition, or the loss of an atom. The only change has been in the arrangement of their chemical equivalents, and this has been sufficient to produce new forms, new states, new properties.

But the solids are not fixed. They are consumed and disappear in the actions of vitality, to be reformed from the plasma of the blood. Yet we do not find the organic matter of the solids escaping. No albumen, or fibrin, or proteine, or gelatin, are found in the excretions. What then is the mode of their elimination? The chemical elements of the organic matter, enter into new combinations, form new substances, new products, that are found in the excretions, principally the urine and the bile. The excretions and the secretions together, give the chemical equivalents of the blood and the solids.

Here are all the evidences of chemical actions. It is true, they surpass all that chemists can, or ever will accomplish. But they are not the less chemical actions. They are appointed to be executed by the living laboratory of organic beings, constructed by the Supreme Artificer of the Universe. The clumsy contrivances of man, never can pretend to imitate processes of such surpassing delicacy.

There is, then, a chemistry of living beings, intimately associated with their vital actions, and necessary to their completion. The chemical actions of life, form the materials, the immediate organic elements, used by the creative force of life, in the construction of the living organism.

Organic chemistry has not, as yet, been an available source of accurate knowledge, applicable to our science. Chemistry had not, until a recent period, reached the height where organic chemistry begins. But organic chemistry has now acquired development and activity. It has taken its position as a department of the physiological sciences, which must depend upon its future movements, so closely are they interwoven, for their progressive advancement. It cannot be neglected without the danger of a charge of culpable ignorance. Organic chemistry has driven a shaft into the depths of organic phenomena, and struck on a rich vein. No one can foresee the importance of the facts it will bring into light, or the changes it may effect. The recent work of Justus Leibig, is the first successful effort in this unexplored department of medical science. It is to be regretted that he has not confined himself to the experimental part of his investigations, and has prematurely indulged in speculation, for which the science is not sufficiently ripe. What he has done is a promise of the rich stores that will be poured out from the exploration of this vein of knowledge. The most skeptical must be startled at the facts, unquestionably established, and the views they open in the vista of our science.

It cannot be doubted, that organic chemistry is the most important of the means that can impart clear, defined, precise ideas of the processes of the organic functions, in health, in disease, and under medicinal influence. When in possession of this positive knowledge, instead of the notions, it must be confessed, too frequently loose and uncertain that now prevail, it is not possible to anticipate the suggestions it will awaken in intelligent minds, as to the means of preserving health, and the remedial agencies adapted to resist and overcome disease.

But let not the pretensions of organic chemistry be carried too far. It can never be looked to, as a basis for the science of medicine, or for more than a partial theory. The limit of its usefulness, is the exposition of the chemical side of the facts of the organic functions, in their natural state and in disease."

The concluding paragraph is apt and forcible.

"I have only again to repeat, that the more clear and distinct the ideas you form of the actions of the living economy, in their proper characters, and of the class to which each belongs, the more confidence you will feel in the correctness of your views and the soundness of your judgments; the more certainty and success will attend your practical skill in devising the means for remedying their defects. The difference between one who comprehends in every aspect the phenomena he has to deal with, and one who knows them imperfectly, is the difference between the clear-sighted and the blind. We may be surprised at what the blind can do, but it is, nevertheless, the doings of the blind. Knowledge is the field of mental vision, and he who refuses to obtain the knowledge in his power, prefers darkness to light. I shall spare no labour in the performance of my duty, and shall illustrate, by numerous drawings and tables, with which I am amply provided, the subjects that can be graphically represented. Do not neglect your duty. Apply resolutely to the work before you, and difficulties will vanish, obstacles be overcome. Willing hearts and ready minds never fail; they command as they merit success."

